# Modeling heavy metal release in the epiphytic lichen *Evernia prunastri*

**DOI:** 10.1007/s11356-021-12620-7

**Published:** 2021-01-28

**Authors:** Andrea Vannini, Luca Paoli, Riccardo Fedeli, Sharon Kwambai Kangogo, Massimo Guarnieri, Stefania Ancora, Fabrizio Monaci, Stefano Loppi

**Affiliations:** 1 Department of Life Science, University of Siena, I-53100 Siena, Italy; 2 Department of Biology, University of Pisa, I-56126 Pisa, Italy; 3 Department of Physics, Earth and Environmental Sciences, University of Siena, I-53100 Siena, Italy

**Keywords:** Air pollution, Bioaccumulation, Biomonitoring, Cu, Environmental recovery, Zn

## Abstract

In this study, the release of Cu^2+^ and Zn^2+^ was investigated and modeled in the epiphytic lichen *Evernia prunastri.* Samples were incubated with solutions containing these metals at ecologically relevant concentrations (10 and 100 μM) and then transplanted to a remote area and retrieved after 1, 2, 3, 6, 12, and 18 months. The results showed that, after 12 months, all samples faced similar metal reductions of ca. 80–85%, but after this period, all the involved processes seem to be no longer capable of generating further reductions. These results suggest that the lichen *E. prunastri* can provide information about environmental improvements after exposure to high or very high pollution levels in a relatively short period of time.

## Introduction

Lichens have long been considered good bioindicators of air quality and a very effective biological tool to evaluate the environmental availability of trace metals (Bačkor and Loppi 2009). The content of heavy metals in lichens, which is known to be proportional to their content in bulk deposition (Loppi and Paoli 2015), is the result of a dynamic process in which both accumulation and release events occur, until an equilibrium with the surrounding environment is reached (Nieboer et al. 1978).

Changes in the metal content of lichens as a consequence of changes (either increases or reductions) in metal environmental availability require some time to occur (called “remembrance time,” see Reis et al. 1999) that is generally assumed to be shorter for accumulation events than for releases. In fact, when lichens face increased heavy metal availability (e.g., when they are transplanted to polluted areas), they may require up to 12 months to reach an equilibrium with the new environment (Godinho et al. 2008; Kularatne and De Freitas 2013; Paoli et al. 2018a), while when metal availability is decreased, lichens require a much longer time, up to 5 years, to release them and reach a new equilibrium with the surrounding environment (Walther et al. 1990; Nieboer and Richardson 1981). Nevertheless, when metals are provided in ionic form dissolved in water, lichens require a much shorter time (from a few minutes to hours) to reach an equilibrium (Loppi et al. 2020; Paoli et al. 2018b), but also in this case, metal ion release requires a longer, although undetermined, time (Loppi et al. 2020). Although the dynamic of accumulation of heavy metals in lichens is well documented (Bačkor and Loppi 2009), information about their release is minimal, and studies dedicated to model the time required by lichens to release the accumulated metals are very scanty.

In this study, the modeling of Cu^2+^ and Zn^2+^ releases provided in ionic form was investigated in the lichen *Evernia prunastri*, with the hypothesis that 18 months are sufficient for “contaminated” thalli to reach an equilibrium with a relatively unpolluted environment.

## Materials and Methods

### Sample collection

Samples of the lichen species *Evernia prunastri* (L.) Ach. were collected in a remote area of the Siena Province (43° 11′ 60″ N, 11° 21′ 33″ E, 310 m a.s.l.), located far from any local pollution source. Lichen thalli were harvested from the branches of *Prunus spinosa* shrubs taking care to collect only those with lobes longer than 5 cm (adult thalli). This species was selected being widely used in biomonitoring studies (Loppi et al. 1998; Vannini et al. 2017) and for its documented ability to accumulate heavy metals (Loppi and Paoli 2015). In the laboratory, lichen thalli were cleaned from any extraneous material (e.g., other lichen species, mosses, and bark residues) using plastic tweezers, washed three times for 5 s with deionized water to remove superficial dust then let air-dry overnight.

### Experimental

The whole lichen pool was randomly subdivided into five batches, each consisting of 12.25 g of lichen material and subsequently treated as follows: four batches were separately incubated (200:50 w:v) for 1 h with Cu^2+^ and Zn^2+^ 10 and 100 μM solutions obtained from chloride salts. The pH of the solutions was adjusted to 5.5 to maximize metal uptake (Chettri et al. 1997). The last batch was incubated with deionized water (pH adjusted to 5.5) and used as control. After treatment, samples were quickly rinsed three times in deionized water to remove metal amounts simply deposited on the surface of thalli and then let air-dry to allow for a possible later metal uptake (Brown and Beckett 1985).

Each batch of samples was randomly subdivided in seven subsets having a weight of 1.75 g from which seven “lichen bags” of approximately 0.25 g were prepared (statistical replicates). The lichen material was loosely wrapped inside plastic net (mesh 1 cm^2^), closing them at the two extremities using plastic wire. One of these seven subsets was air-dried overnight in a climatic chamber at 16 °C and 55% RH (residual water content < 10%) and then stored at −20 °C until analyses and later used as a starting point for the evaluation of the elemental release (month 0). Treatment concentrations, selected in order to generate metal uptake in lichens (Paoli et al. 2018b), are within the range of ecologically relevant metal concentrations in polluted environments (Chettri et al. 1997).

### Lichen exposure

The lichen bags were exposed in a new and relatively unpolluted environment located in a rural area of the Grosseto Province (42° 41′ 23″ N, 11° 20′ 05″ E) at ca. 2 m from the ground, during October 2017. This site was selected being protected from vandalism and to allow for an easy check of the samples during this long-lasting experiment. Samples were retrieved after 1, 2, 3, 6, 12, and 18 months from the transplantation. In the laboratory, samples were removed from bags, air-dried overnight in a climatic chamber at 16 °C and 55% RH and then stored at −20 °C until analyses. Table 1 shows the monthly rainfall at the exposure site during the study period.

**Table 1:** Total monthly rainfall at the exposure site during the study period

Time (months)	Month	Year	Rain (mm)
1	October	2017	3.4
2	November	2017	25.4
3	December	2017	58.6
4	January	2018	59.6
5	February	2018	141.8
6	March	2018	192.8
7	April	2018	30.6
8	May	2018	135.8
9	June	2018	102.2
10	July	2018	3
11	August	2018	58.6
12	September	2018	6.8
13	October	2018	94.6
14	November	2018	129.2
15	December	2018	45.8
16	January	2019	78
17	February	2019	64.2
18	March	2019	6.8

### Chemical analysis

Prior to mineralization, each sample was pulverized in liquid nitrogen with a ceramic mortar and pestle. Approximately 200 mg of lichen powder was mineralized in a MW digestion system (Milestone Ethos 1) with 7 mL of HNO_3_ and 3 mL of H_2_O_2_ using hermetic Teflon vessels at 130 °C and high pressure (100 bar). Depending on the concentration, the mineralized and diluted solutions (up to 50 mL) were analyzed either by inductively coupled plasma atomic emission spectrometry (ICP-AES, Optima 5300 dv; Perkin Elmer) or by transversely heated graphite furnace (THGA) with longitudinal Zeeman-effect background corrector atomic absorption spectrometry (AAnalyst 800; Perkin Elmer). The results are expressed on a dry weight basis (μg g^−1^ dw). Analytical quality was checked by analyzing the Standard Reference Material IAEA-336 “lichen,” which indicated recoveries in the range 97–101%. The precision of the analytical procedure was estimated by the coefficient of variation of four replicates that was within 5% for both elements.

### Statistics

To account for possible temporal variations in the atmospheric deposition at the exposure site, results were reported as ratio between exposed samples to their respective temporal control (exposed-to-control, EC ratio). Statistically significant differences between exposed and control samples as well as variations of the EC ratio across time were checked using the Wilcoxon signed-rank test, applying a correction for multiple testing according to Benjamini and Hochberg (1995).

Non-linear regression analysis (exponential, power, and logarithmic) was run to model the relationship between EC values and exposure time. The best fitting was sought comparing *R*^2^ and AIC (Akaike Information Criterion) values. Equation coefficients were used to model the release rates of Cu and Zn (EC month^−1^) by their derivatives. All calculations were run using the free software R (R Core Team 2020).

## Results

**Fig. 1:**
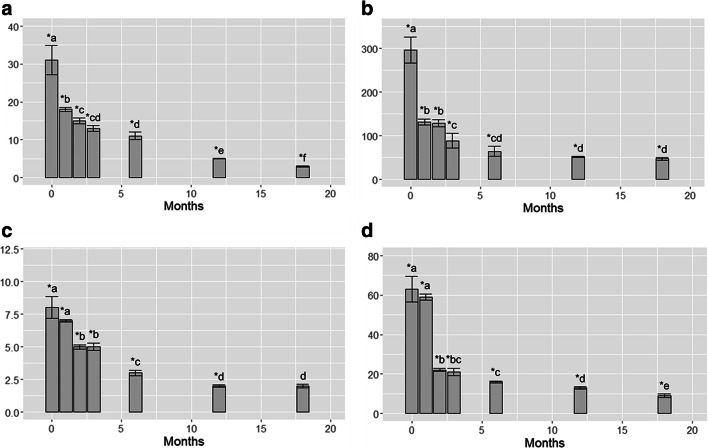
Accumulation values (EC ratio) (mean ± SE) in samples of *E. prunastri* treated with Cu 10 μM (**a**), Cu 100 μM (**b**), Zn 10 μM (**c**), Zn 100 μM (**d**), and transplanted in a clean environment for 0, 1, 2, 3, 6, 12, and 18 months. The asterisk indicates statistically significant differences between treated and control samples (*p* < 0.05). Different letters indicate statistically significant differences between EC values across time (*p* < 0.05)

During the exposure, control samples showed Cu and Zn concentrations (5.6 ± 0.6 and 40.2 ± 3.8 μg g^−1^, respectively) not exceeding those (6 ± 0.6 and 43 ± 4 μg g^−1^) reported for the lichen *E. prunastri* from background areas (Cecconi et al. 2019). Results of the temporal release of Cu and Zn from samples of *E. prunastri* after their transplantation in the remote environment are shown in Fig. 1. Statistically significant (*p* < 0.05) releases of Cu were evident already after 1 month. In detail, after the treatment with 10 μM samples showed a decrease by 40% after 1 month, by about 65% after 6 months and finally by about 91% after 18 months. Continuous statistically significant (*p* < 0.05) decreases across time were observed. After the treatment with 100 μM, samples showed similar decreasing trends, with reductions approximately by 56% after 1 month, by 80% after 6 months and by 84% after 18 months; no statistical (*p* > 0.05) differences were detected from 6 to 18 months from the transplantation.

Both Zn exposures showed very similar release trends after transplantation into the new environment irrespective of the treatment. Specifically, Zn showed statistically significant (*p* < 0.05) releases starting after 2 months, with decreases in the range 39% for samples treated with 10 μM and 65% for those treated with the 100 μM. After 6 months, samples exposed to 10 μM and 100 μM showed significant (*p* < 0.05) reductions by 69% and 75%, respectively, while after 18 months, a similar average reduction by ca. 83% was evident. Statistically significant (*p* < 0.05) differences between 6 and 18 months were observed for both exposures.

After 18 months from the transplantation, only samples exposed to Zn 10 μM did not show any statistically significant (*p* > 0.05) difference compared with control samples.

**Fig. 2:**
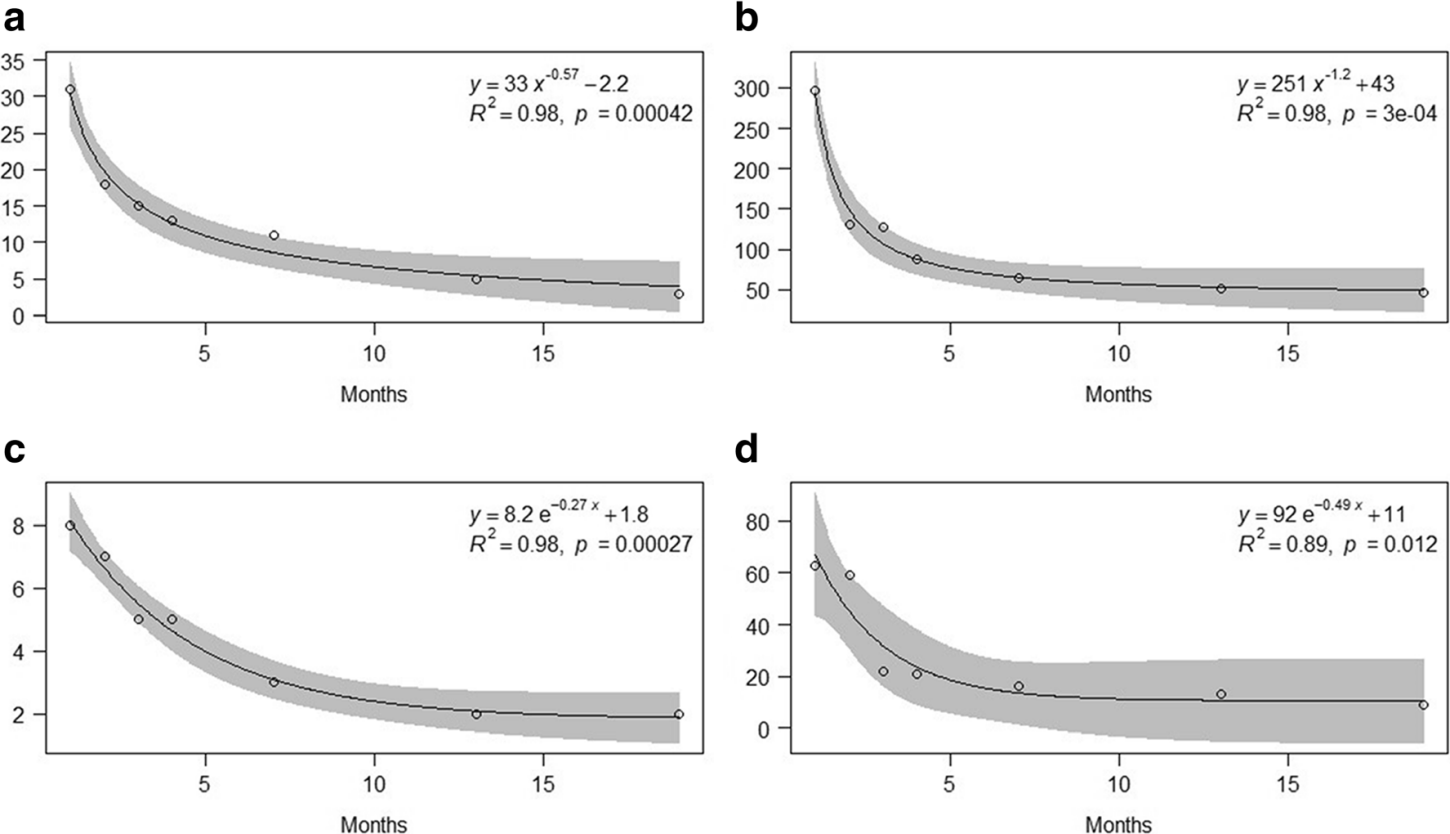
Exponential and power regressions of accumulation values (average of the EC ratio) across time in samples of *E. prunastri* treated with Cu 10 μM (**a**), Cu 100 μM (**b**), Zn 10 μM (**c**), Zn 100 μM (**d**), and transplanted in a clean environment for 0, 1, 2, 3, 6, 12, and 18 months. The gray area indicates 95% confidence interval

**Fig. 3:**
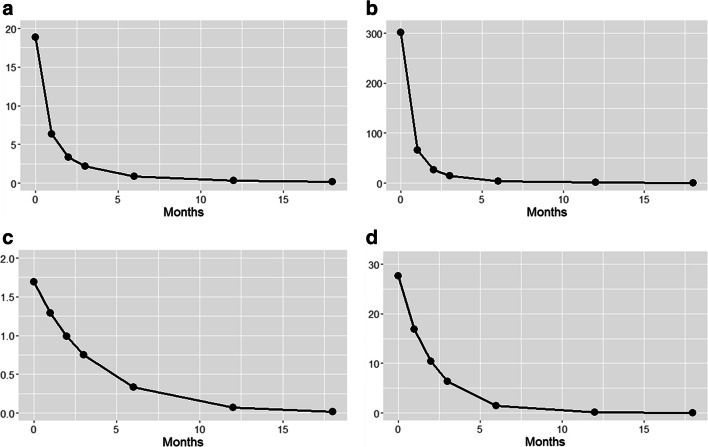
Release rates (EC month^−1^) in samples of *E. prunastri* treated with Cu 10 μM (**a**), Cu 100 μM (**b**), Zn 10 μM (**c**), Zn 100 μM (**d**) after their transplantation in a clean environment for 0, 1, 2, 3, 6, 12, and 18 months

Samples treated with Cu showed the best relationship between EC values and transplantation time with the power regression model, while those exposed to Zn with the exponential regression model (Fig. 2). The results of the calculated release rates for each sample at all exposure time are presented in Fig. 3. Samples treated with Cu showed abrupt decreases in their release rates already after 1 month from the transplantation and irrespective of the treatment, while those exposed to Zn showed more gradual reductions across time. Starting from 12 months from the transplantation, the release rates reached almost constant values.

## Discussion

This study investigated the release dynamics of accumulated ionic metals, namely, Cu^2+^ and Zn^2+^, in thalli of the lichen *E. prunastri* after their transplantation into a relatively unpolluted environment. Our results indicate relative element releases, expressed in terms of EC ratios, across time, with losses already evident after the first 1–2 months from the transplantation; moreover, after 18 months, all samples showed similar 85% reductions. Only samples exposed to Zn 10 μM reached background values, while the others still retained important amounts of metals, with EC values suggestive of a severe accumulation (Cecconi et al. 2019). We argue that the complete release of Zn from lichen samples treated with 10 μM solutions of this metal may be related to its limited accumulation after the treatment (month 0), less than 75% of the accumulated Cu at the same exposure time and level. This result is further confirmed by the closer approach to background values for samples treated with Zn 100 μM than for those treated with the same amount of Cu.

A decrease in the elemental content in lichens may occur following different mechanisms, as in the succession of wetting and drying cycles (Nieboer and Richardson 1981), the accumulation of other metals with higher affinity for the extracellular bindings sites, leading to the displacement of weaker ions (Paoli et al. 2018b), or the biomass increase (growth) of the thalli which may cause dilutions in their respective contents (Bargagli and Mikhailova 2002). Nevertheless, metal release from lichens may also occur following their excretion onto the thallus surface after their complexation by oxalates and secondary compounds (Sarret et al. 1998), which may subsequently be dislodged by the action of rainfall, similar to insoluble particulate matter deposition (Brown and Brown 1991). We may argue that a fast Cu release from lichen thalli may be due to this latter mechanism, which acts to limit the bioavailability of this metal as much as possible, being Cu highly toxic for lichens at elevated concentrations (Bačkor and Loppi 2009).

Information about the time that lichens require to accomplish the complete release of their accumulated metal content is very limited; nevertheless, consistently with our results, field studies indicated that lichens may require more than 18 months to release their accumulated metals following environmental improvements. Specifically, Paoli et al. (2018a) reported that samples of the lichen *Flavoparmelia caperata* collected from the surroundings of a landfill and transplanted for 1 year into an unpolluted site, faced only slight decreases of ca. 30%, and speculated that at least 2 years are necessary for lichens exposed to high amounts of depositions to reach background concentrations. Similar results (half memory time of 200 to 600 days) were calculated for thalli of *Parmelia sulcata* transplanted nearby a power plant (Reis et al. 1999) and observed (2–4 years) in thalli of *Parmotrema praesorediosum* and *Ramalina stenospora* after the stop of industrial emissions (Walther et al. 1990). Moreover, the results reported by Walther et al. (1990) for *R. stenospora* are consistent with those observed in our study for samples treated with Zn 10 μM, with similar concentrations of ca. 100 μg/g and a similar time of about 1 year to reach steady background values. On the other hand, samples of *F. caperata* transplanted for 6 months in a polluted area and then brought back to the reference site for 3 months showed the complete release of the previously accumulated Cu and Zn (Godinho et al. 2011).

The results of the release rates suggest that after 12 months any further metal reduction is negligible. We argue that most of the remaining non-released fraction (about 15–20% of the accumulated metals) may be bound to the cell wall exchange sites, as highlighted by Loppi et al. (2020). This fraction can be regarded as the “memory” of the past pollution event (Reis et al. 2002; Boulyga et al. 2003; Dalvand et al. 2016). These results have important implications for biomonitoring since, if on the one side confirm that lichens do reflect an averagely integrated time period of exposure, on the other side they indicate that under certain circumstances they may not at all reflect the current pollution level at a site.

## Conclusions

Our results allowed to accept the working hypothesis that 18 months are a sufficient amount of time for lichen samples exposed to ecologically relevant concentrations (10 and 100 μM) of Cu and Zn ions to reflect an improved environmental simulation. After 12 months, all samples showed similar metal reductions of ca. 80–85%, but after this period, all the involved processes seem to be no longer capable of generating further reductions. These results suggest that the lichen *E. prunastri* can provide information about environmental improvements after exposure to high or very high pollution levels in a relatively short period of time. Nevertheless, the temporal dimension of the outcomes of transplant studies using this species, usually involving exposure periods of 1–6 months, has to be evaluated carefully.

## Data Availability

The datasets used and/or analyzed during the current study are available from the corresponding author on reasonable request
